# Dietary inclusion of nano-phosphorus improves growth performance, carcass quality, and growth-related traits of Nile tilapia *(Oreochromis niloticus)* and alleviates water phosphorus residues

**DOI:** 10.1007/s10695-023-01199-0

**Published:** 2023-05-04

**Authors:** Anwar Elamawy, Elsayed Hegazi, Eldsokey Nassef, Tarek K. Abouzed, Abeer G. Zaki, Taha Ismail

**Affiliations:** 1 Department of Nutrition and Clinical Nutrition, Faculty of Veterinary Medicine, Kafrelsheikh, Egypt; 2 Department of Biochemistry, Faculty of Veterinary Medicine, Kafrelsheikh University, Kafrelsheikh, Egypt; 3 Biotechnology Department, Animal Health Research Institute, Giza, Egypt

**Keywords:** Nile tilapia, Nano-phosphorus, Nanoparticles, Water quality, Growth-related genes

## Abstract

Supplementation of phosphorus nanoparticles is a promising strategy to reduce water pollution, improve phosphorus concentration in fish diet, and provide better production quality. We used 300 fingerlings of Nile tilapia that were randomly distributed into 3 groups; each one was attributed to 5 replicates of 20 fish per aquarium with initial weight (gm) (156 ± 1.25). The first diet contained traditional Di-calcium phosphate (D-group), the second supplemented with phosphorus nanoparticles in a dose equal to the previous conventional one (N-D group), and the last one included with phosphorus nanoparticles with the half dose of the conventional phosphorus group (1/2 N-D group). After 3 months of feeding, the N-D group showed the best growth performance including its feed conversion ratio (FCR), feed intake (FI), or body weight gain (BWG). Furthermore, the growth-related gene expression findings considering growth hormone receptor (GHR) and insulin-like growth factor-1 (IGF-1) were upregulated as well. Moreover, whole body chemical composition revealed higher Fe, Zn, P, and crude protein level in the N-D group than the other two groups. Lipoprotein lipase (LPL) and fatty acid synthetase (FAS) mRNA expression showed a significant increase in 1/2 N-D and N-D groups compared with the control group. To sum up, using of nano-phosphorus particles improved the growth rate and immunity response of Nile tilapia, besides decreasing water pollution.

## Introduction

In comparison with other nutrients, studies regarding the minerals in fish and crustaceans are scarce. Furthermore, there is much less information available about the mineral requirements of aquatic species compared to terrestrial animals. Interestingly, fish has the ability not only to absorb minerals from the aquatic medium in which they live but also from their diet.

Phosphorus is represented in a wide range of organic phosphates, such as deoxyribonucleic acid (DNA), phospholipids, coenzymes, nucleotides, and ribonucleic acid (Bueno et al. 2019; Fraser et al. 2019). Inorganic phosphates are also used as an important buffer for preserving the pH for intracellular and extracellular fluids in a normal state (Zubay 1983). Increasing dietary phosphorous coincided with expanding add up to phosphorous within the liver and lessening the feed conversion ratio (Luo et al. 2010; Liu et al. 2022). Interestingly, phosphorus supplementation in fish diets is usually very important because of its limited utilization by fish and, meanwhile, its presence in water. In contrast to calcium, phosphorus availability in natural water is typically very low (Boyd 1981). As a result, sufficient quantity of phosphorus absorption from freshwater and saltwater is limited (Lall 1991), so supplementation of phosphorus is so important for both fish and shrimp. Phosphorus deficiency in the diet impairs metabolism, resulting in diminishing of growth and feed utilization. Suboptimal phosphorus intake also causes less mineralization of hard tissues which usually leads to some skeletal deviations or malformations (Sugiura et al. 2004). In young fish, scales phosphorous content appears to be the most sensitive indicator of phosphorous deficiency (Lall et al. 2021). Fish species’ phosphorus needs have ranged from 0.3 to 1.5 of the diet percent (Lall 2002).

The impact of excreted phosphorus on the eutrophication of water has resulted in a significant amount of research papers on phosphorus as a nutrient, in recent years. Intensification in aquaculture led to a deleterious effect on water system due to eutrophication, which in turn has serious effects on aquatic ecosystems including disruption of natural biogeochemical cycling, anoxia, and destruction of biodiversity (Lake et al. 2000; Compton et al. 2011). Sediment-bound phosphorus is a well-known driver of nutrient enrichment in freshwater ecosystems across the globe (Correll 1998). Years have passed with aiming to reduce phosphorus excretion. The sensitive indicator of dietary phosphorus metabolism in fish has been known through the urinary phosphorus concentration (Sugiura et al. 2000). Various dietary manipulations, such as lowering total dietary phosphorus or increasing phosphorus availability in the diet by adding phytases or other additives such as citric acid, have shown to reduce urinary and fecal phosphorus excretions (Gatlin and Li 2008; Green et al. 2002; Sugiura et al. 1998). Nano form of minerals is often ranged between 1 and 100 nm, which improves the chance of absorption and makes it available through different body tissues and fluids. Additionally, small size and large surface characters of nanoparticles have a positive effect on the induction of their biological and physiological ability. Nano form of minerals reduces mineral antagonism in the intestine, decreasing excretion of those minerals; thus, it reduces environmental pollution (Gopi et al. 2017). To our best knowledge, this research is the first scientific document reporting effects of phosphorous nanoparticles on Nile tilapia growth performance and growth-related genes.

Our work was designed to investigate, for the first time, the potential impact of phosphorus nanoparticles instead of conventional phosphorus, which is predicted to increase not only the production and growth performance of Nile tilapia but also reduce the environmental pollution of water, phosphorus concentration in fish diet, cost, and pollution of water.

## Material and methods

### The ethical approval

The experimental procedures were approved and performed following the Veterinary Medicine Faculty of Kafrelsheikh University, Egypt, guidelines about the animal care and ethics committee.

### Diet preparation

Three types of isocaloric and isonitrogenous diets were formulated to fulfill Nile tilapia requirements (NRC 2011), as presented in Table 1. The basal dietary available phosphorous was 0.25%. So, the fish still require 0.25% supplemental phosphorous to meet their requirements according to NRC (2011). The first diet has conventional di-calcium phosphates at a level of (0.25%) phosphorus (D-group), the second supplemented with our phosphorus nanoparticle prepatation at phosphorus level equal to that of D-group (0.25%) (N-D group), and the last one was prepared for phosphorus nanoparticles giving half dose (0.125%) of the conventional phosphorus group (1/2 N-D group). Then, all components were passed through a 1 mm sieve and well mixed. Then, oil was added while stirring continuously in a slow technique. The addition of distilled water to the ingredients gradually until soft dough content formed. The diets were pelleted and dried at 60 °C temperature for 2 h in a hot air oven. Preparation of phosphorus nanoparticles had been done in Lab. of Nanotechnology, Faculty of Science, Kafrelsheikh University. P-nanoparticles (NPs) were purchased from the market (Sigma-Aldrich) and analyzed by XRD and TEM to confirm their crystalline structure and size (Iqbal et al. 2014; Ramamurthy et al. 2013).

**Table 1:** Experimental diets and their chemical composition^*^

Item (%)	D-group	N-D group	1/2N-D group
Fish meal	5.55	5.55	5.55
Corn gluten meal	5.5	5.5	5.5
De-hulled soybean meal	44.5	44.5	44.5
Corn grains	34.9	34.9	35.57
Whole linseed	5	5	5
Fish oil	1	1	1
Soybean oil	1	1	1
Conventional Di-calcium phosphate	1.35	0	0
Nanoparticles Di-calcium phosphate	0	1.35	0.68
Limestone	0.5	0.5	0.5
Salt	0.5	0.5	0.5
Premix^**^	0.2	0.2	0.2
Crude protein (%)	30.49	30.53	30.33
Ether extract (%)	6.63	6.51	6.61
Ash%	4.77	4.43	4.22
Crude fiber (%)	4.75	4.65	4.52
Digestible energy (Kcal/Kg)	2950	2940	2939

^*^The basal dietary phosphorous was 0.25%. D-group supplemented with 1.35% conventional di-calcium phosphate which provides 0.25% phosphorous. N-D group supplemented with 1.35% Nanoparticles di-calcium phosphate which provides 0.25% phosphorous. 1/2N-D group supplemented with 0.68% Nanoparticles di-calcium phosphate which provides 0.125% phosphorous

^**^Premix (Egypt pharma): each 1 kg composed 1000,000 IU Vit. A; 200,000 IU Vit. D3; 10,000 mg Vit. E; 2000 mg K3; 4000 mg B1; 4000 mg B2; 4000 mg B6; 4 mg B12; 20,000 mg niacin; 1000 mg folic; 20 mg biotin; 10,000 mg pantothenic acid; 1000 mg copper; 1 mg cobalt; 1000 mg iodine; 100 mg selenium; 100,000 mg iron; 10,000 mg manganese; 30,000 mg zinc; calcium carbonate ad to 1000 gm

### Fish management and experimental design

Three hundred Nile Tilapia fingerlings, with initial weight (gm) (156 ± 1.25), were kept in a tank for 1 week for adaptation. Then, fingerlings of homogenous size were haphazardly conveyed into 15 glass aquaria (60 × 70 × 100) cm, each five of them representing a single group (*n* = 20). Glass aquaria were equipped with continuous oxygen source with manual 40% daily water change using a syphon. Testing and recordation of water temperature, pH, dissolved oxygen, and ammonia were recorded daily and reported as follows: 25 ± 1.7 °C, 6 ± 0.02 mgL^−1^, 6.1 ± 0.2, and > 0.1 mg, respectively. The experiment lasted for 3 months. Feeding was in every day two times at 8.00 morning and 3.00 p.m. until satiation, and it was nearly about 5% of fish weight.

### Feed, fish body, and water analysis

The chemical composition of both feed ingredients and fish body was analyzed following the standard methods (AOAC 2010). Sample was heated in an oven with hot air at 105 °C till constant weights to estimate the dry matter content. Crude protein content was determined after acid digestion using Kjeldahl’s apparatus (*N* × 6.25). Crude lipid was determined using the Soxhlet apparatus; meanwhile, ash percentage was measured through incinerating the samples at 550 °C temperature for 6 h.

Fe, P, Ca, and zinc were measured using atomic absorption spectrophotometer supported with transversely heated graphite (Younglin AAS 8020, Korea) (Elia et al. 2011).

### Growth and feed utilization parameters

At the end of the feeding trial, all fish were weighed to determine body weight gain (BWG), feed intake (FI), and feed conversion ratio (FCR) following those equations:

$$\mathrm{BWG}=\mathrm{final}\;\mathrm{body}\;\mathrm{weight}\;\left(\mathrm g\right)-\mathrm{initial}\;\mathrm{body}\;\mathrm{weight}\;\left(\mathrm g\right)$$

$$\mathrm{FCR}=\mathrm{feed}\;\mathrm{intake}\;\left(\mathrm g\right)/\mathrm{weight}\;\mathrm{gain}\;\left(\mathrm g\right)$$

$$\mathrm{FI}\left(\mathrm g\;\mathrm{fish}^{-1}\;{90\mathrm{days}}^{-1}\right)=\left(\mathrm{Dry}\;\mathrm{diet}\;\mathrm{given}-\mathrm{Dry}\;\mathrm{remaining}\;\mathrm{diet}\;\mathrm{recovered}\right)/\mathrm{No}.\;\mathrm{of}\;\mathrm{fish}$$

### Blood biochemistry

Blood samples were collected at the end of the experiment from the caudal vein of 5 fish per aquarium for blood sample pooling, centrifuged for 10 min at 3000 rpm to get the serum, and then kept at − 20 °C for other biochemical tests. The serum albumin conc. was measured calorimetrically as previously reported by Doumas et al. (1971). Liver enzymes activity (SGPT and SGOT) were performed as prescribed by Reitman and Frankel (1957). Creatinine and serum triglycerides, cholesterol, low-density lipoprotein (LDL), and high-density lipoprotein (HDL) were assessed using a biochemical auto analyzer (Johnson et al. 1999; Stavros, et al. 2006).

### RNA extraction and reverse transcription (RT)

**Fig. 1:**
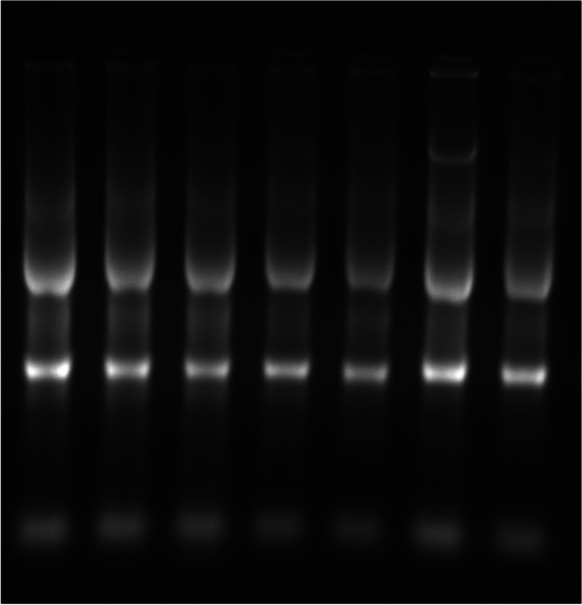
Ethidium bromide-stained agarose gel showing intact extracted RNA from 7 representative samples

Liver samples were prepared for total RNA extraction using easy-RED total RNA extraction kits (iNtRON Biotechnology, Incorporation Kits Cat No, 17,063), following the manufacturer’s guidelines. Six liver samples were obtained from each treatment and stored at − 80 °C until use. The quality of RNA was assured by electrophoresis using agarose gel 1%. As seen in Fig. 1, the intact and high integrity of RNA was determined by the presence of highly sharp and prominent ribosomal RNA bands (18-S and 28-S) and tRNA bands. Total RNA (2 μg) was revering transcribed to the complementary cDNA using (TOPscript ^TM^RT DryMIX, Cat #RT220).

### Real-time PCR-quantitative (qRT-PCR)

Primers of specific genes for growth regulatory genes GHR and IGF and fat metabolism genes LPL and FAS were used for qPCR assay. The housekeeping gene was β-actin. Each gene contained 10 μl of SensiFast™ SYBR Lo-Rox master mix (Bioline, UK); the reaction mixture for each was 2 μl of cDNA and 0.5 μM of each primer. Tests were made in duplicates by the Mx3005P real-time PCR system. Steps of the amplification parameters were done as follows: denaturation at 95 °C for 15 min as the initial step, followed by 40 cycles at 95 °C for 15 min. The step of annealing was done for 1 min at gene-specific annealing temperatures according to Table 2. When the dissociation curves were analyzed for all tested genes, only one peak was shown at a specific melting temperature them, indicating specifically amplified PCR products, the 2 − ΔΔct was the method used for evaluation of the fold change for each gene according to Livak and Schmittgen (2001).

**Table 2:** Primers pairs sequences used in the quantitative real-time PCR reactions

Gene	Primer sequence (5'-3')	Annealing temp	References (accession no.)
*FAS*	F: TGAAACTGAAGCCTTGTGTGCC R: TCCCTGTGAGCGGAGGTGATTA	59 °C	(Aanyu et al. 2018) GU433188
*LPL*	F: TGCTAATGTGATTGTGGTGGAC R: GCTGATTTTGTGGTTGGTAAGG	60 °C	(Aanyu, Betancor et al. 2018) NM_001279753.1
*IGF-I*	F: GTTTGTCTGTGGAGAGCGAGG R: GAAGCAGCACTCGTCCACG	60 °C	(El-Kassas et al. 2020) Y10830.1
*GHR1*	F: CAGACTTCTACGCTCAGGTC R: CTGGATTCTGAGTTGCTGTC	60 °C	(El-Kassas, Abdo et al. 2020) AY973232.1
*β-actin*	F: CAGCAAGCAGGAGTACGATGAG R: TGTGTGGTGTGTGGTTGTTTTG	60 °C	(El-Naggar et al. 2021) XM_003455949.2

### Statistical method

The analysis of variance (ANOVA) is the statistical analysis method that was performed. The significant difference among means at *P* < 0.05 (means ± SEM). Duncan’s multiple comparisons made after significant differences were existed. The SPSS program has been used to put all the statistics on the computer with it (SPSS 2004).

## Results

### Characterization of nano-phosphorus particles

**Fig. 2:**
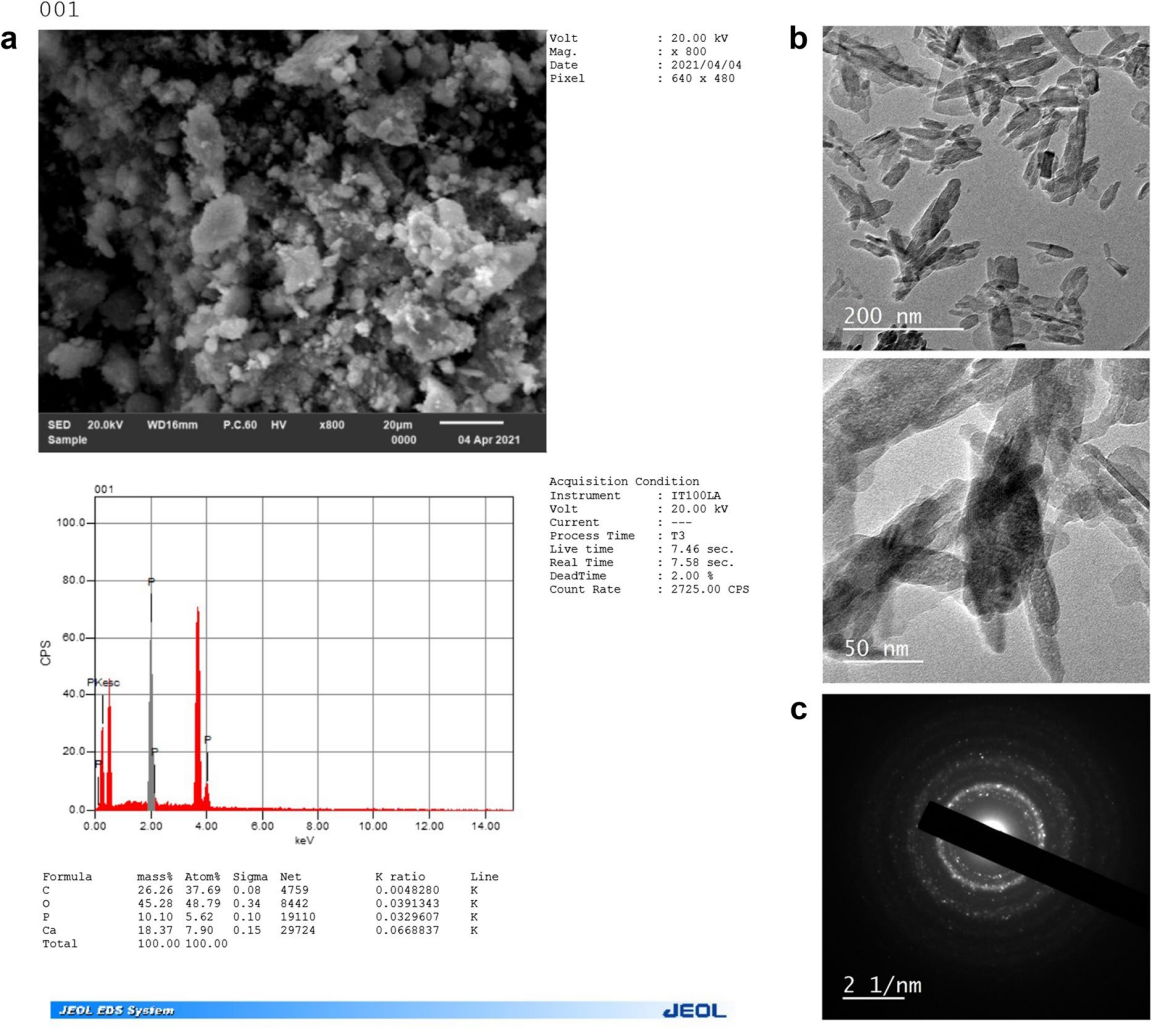
(**a**) Characterization of nano-phosphorus by transmission electronic microscopy (TEM) image and energy-dispersive X-ray spectroscopy (EDX) image. (**b**) Transmission electron microscopic (TEM) view of phosphorus Nanoparticles, with magnified form 50 nm scale bar, 200 nm scale bar. (**c**) X-ray diffraction (XRD) techniques

The sample is surely nano crystalline in nature and that was confirmed by the XRD pattern as it matches clearly with the standard phosphorus powder of phosphorus nanoparticles (Fig. 2A, C). In Fig. 2A, all the synthesized phosphorus nanoparticles (P-NPs) showed a homogeneous distribution by transmission electron microscope (TEM). In Fig. 1B, TEM confirms the shape and size of P-NPs, and TEM image justifies the broken glass or needle shape form of phosphorus-NPs. The above results of TEM confirm that they were pure nanoparticles. The crystal structure and the phase composition of phosphorus nanoparticles were assured through X-ray diffraction (XRD) techniques as shown in Fig. 2C.

### Growth performance

As shown in Table 3,1/2 N-D and control groups showed the same feed intake (FI) response (*p* ˃ 0.05); meanwhile, the N-D group reported the lowest FI values. Moreover, the best FCR was noticed in the N-D group (*p* ≤ 0.05). Final weight (FW) and body weight gain (BWG) recorded insignificantly differed between groups (*p* ˃ 0.05).

**Table 3:** Growth performance of Nile tilapia fed conventional dietary phosphorus and nano-phosphorus for 12 weeks

	D-group	N-D group	1/2 N-D group
IW (g)	156.00 ± 1.15	157.67 ± 3.18	157.33 ± 0.67
FW (g)	349.00 ± 13.20	374.00 ± 7.37	345.33 ± 14.72
FI (g)	285.67 ± 1.45^a^	253.33 ± 1.20^b^	286.33 ± 2.33^a^
BWG (g)	193.00 ± 14.29	216.33 ± 10.37	188.00 ± 14.73
FCR	1.49 ± 0.12^a^	1.17 ± 0.07^b^	1.52 ± 0.10^a^

Different lowercases indicate significant differences (*P* < 0.05)

### Biochemical parameters

Table 4 summarized the results of the biochemical analysis. SGOT liver enzyme showed a significant increase in the D-group and 1/2 N-D group; meanwhile, it reduced in the N-D group. Albumin significantly increased in the N-D group followed by the D-group, and then the 1/2 N-D group). Lipid profile and creatinine kidney enzyme show no significant difference between all groups (*p* ˃ 0.05).

**Table 4:** Biochemical parameters of Nile tilapia fed conventional dietary phosphorus and nano-phosphorus for 12 weeks

	D-group	N-D group	1/2 N-D group
SGPT(U/L)	32.18 ± 0.61	31.84 ± 0.44	32.47 ± 0.39
SGOT(U/L)	225.67 ± 9.94^a^	156.67 ± 4.81^b^	201.33 ± 8.69^a^
Creat. (mg/dl)	0.58 ± 0.15	0.85 ± 0.16	0.57 ± 0.12
Albumin (g/dl)	0.93 ± 0.08^ab^	1.19 ± 0.10^a^	0.82 ± 0.07^b^
Cholesterol (mg/dl)	109.33 ± 7.88	128.33 ± 15.89	111.33 ± 4.49
Triglycerides(mg/dl)	189.33 ± 21.46	176.33 ± 25.89	177.33 ± 13.53
HDL (mg/dl)	36.67 ± 4.41	35.67 ± 4.26	41.33 ± 2.60
LDL (mg/dl)	35.00 ± 8.02	57.67 ± 17.68	35.00 ± 8.19

Different lowercases indicate significant differences (*P* < 0.05)

### Body chemical composition

As shown in Table 5, a significant increase in crude protein was noticed in the N-D group than in other groups. There was also a significant increase in the percent of carbohydrates in the 1/2 N-D group followed by the D-group, leaving the N-D group in the bottom. Crude fat (EE) showed a significant increase in the D-group and N-D group than the 1/2 N-D group (*p* ≤ 0.05). There is no clear difference between groups in other parameters such as ash, moisture, and DM percent.

**Table 5:** Carcass chemical composition of Nile tilapia fed conventional dietary phosphorus and nano-phosphorus for 12 weeks

	D-group	N-D group	1/2 N-D group
DM%	24.02 ± 0.07	24.47 ± 0.16	24.44 ± 0.16
Moisture%	75.72 ± 0.21	75.24 ± 0.03	75.35 ± 0.28
CP%	54.94 ± 0.85^b^	60.60 ± 0.47^a^	55.57 ± 0.39^b^
EE%	12.29 ± 0.10^a^	11.98 ± 0.07^a^	11.02 ± 0.12^b^
Ash%	15.98 ± 0.15	15.44 ± 0.24	15.48 ± 0.13
CHO%	14.55 ± 0.21^b^	11.36 ± 0.16^c^	17.09 ± 0.11^a^
Fe (mg/dl)	225.18 ± 4.33^b^	325.43 ± 24.81^a^	223.83 ± 1.48^b^
Zn (mg/dl)	211.88 ± 11.45^ab^	236.28 ± 10.10^a^	192.30 ± 8.10^b^
P (mg/dl)	17,226.50 ± 131.50^c^	21,346.83 ± 191.07^a^	19,681.17 ± 43.83^b^
Ca (mg/dl)	15,831.17 ± 647.04^b^	18,479.17 ± 905.25^ab^	19,502.10 ± 873.65^a^

Different lowercases indicate significant differences (*P* < 0.05)

Analysis of body mineral composition is expressed in Table 5. Fe element was significantly increased in the N-D group than in other groups. Zn showed a noticed increase in the N-D group followed by the D-group compared with the 1/2 N-D group which had a slight decrease. P element was significantly increased in the N-D group compared with the other experimental groups. Ca body content showed an insignificant difference in the 1/2 N-D group and N-D group; meanwhile, it diminished in the D-group.

### Ca and P in water

As presented in Table 6, phosphorus excretion was significantly decreased in the water of the N-D group followed by the 1/2 N-D group; meanwhile, the highest excretion was noticed in the D-group.

**Table 6:** Ca and P in water of Nile tilapia fed conventional dietary phosphorus and nano-phosphorus for 12 weeks

	D-group	N-D group	1/2 N-D group
Ca (mg/L)	48.90 ± 0.76	51.43 ± 0.90	50.19 ± 0.97
P (mg/L)	0.76 ± 0.14^a^	0.30 ± 0.11^c^	0.40 ± 0.05^b^

### Gene expression measurements

**Fig. 3:**
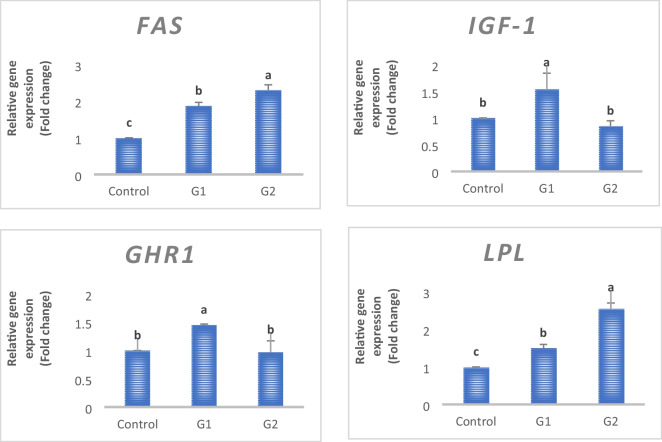
The relative expression of GHR, IGF, FAS, and LPL in the tilapia liver fed diet supplemented with 100% and 50% phosphorus nanoparticles in G2 and G3. Values are means ± SEM. Different lowercase letters show statistical significance at *P* < 0.05

The relative mRNA expression of growth regulatory genes such as growth hormone receptor (*GHR*) (Fig. 3) and insulin-like growth factor (*IGF-1*) (Fig. 3) was altered by nano-phosphorus partials supplementation in the diets. The N-D group showed a significantly upregulated expression of GHR and IGF more than other groups (*p* < 0.05). Regarding to the fat metabolism-related genes (*FAS* and *LPL)*, the 1/2 N-D group, which was supplemented with a half dose of nano-phosphorus, showed the highest mRNA expression of both genes followed by the N-D group.

## Discussion

Organic phosphorus is an important component of several vital mechanisms, such as the formation of nucleotides, coenzymes, phospholipids, hard tissue, deoxyribonucleic acid (DNA), and ribonucleic acid. Inorganic phosphates act as important buffers to keep the pH of fluids either intra or extracellular (Zubay 1983).

Many attempts have been made to reduce the urinary and fecal phosphorus excretion either by reducing phosphorus in the diet or increasing the availability of phosphorus in the diet by adding phytases or other additives such as citric acid (Green et al. 2002; Sugiura et al. 1998; Gatlin and Li 2008).

In our experiment, we tried to reduce the loss of phosphorous in the water and increase its gut absorption by tilapia fish to monitor the extent of its impact on growth and physiological state of Nile tilapia. This could diminish the cost of feeding through increasing its bioavailability and reducing water pollution with phosphorous, which serves as a world’s trend in confronting the acute water problems. Therefore, in the present work, we use the phosphorous in the form of nanoparticles instead of using the traditional source, which was proved to have the ability to inhibit mineral antagonism in the intestine, decreasing there wasting in water (Gopi et al. 2017). Meanwhile, the traditional phosphorus particles are wasted in the water without sufficient absorption by the fish, coincided with a deleterious impact on water quality (Hafez et al. 2019; Gopi et al. 2017; Swain et al. 2015).

Our findings revealed that the N-D group showed the best growth performance including BWG, FI, FCR, and FW. Interestingly, using of minerals in nano form has a great effect on growth performance as assumed by Swain et al. (2015) and Behera et al. (2014). This also matched with the words of Gopi et al. (2017) who reported that the response to nano form minerals has higher bioavailability than conventional ones. Hina et al. (2015) found that using of nano-zinc oxide (nZnO) improved the growth performances of juvenile C. idellain more than other inorganic conventional forms. In accordance with Tawfik et al. (2017), feeding of Nile tilapia fish on nano zinc oxide promoted growth performance more than other inorganic conventional sources. Ghazi et al. (2021) had pointed to the same meaning as they reported a clear enhancement in weight gain and final body weight in tilapia fish treated with nano-selenium and nano zinc synergistically. Additionally, the best BWG and FCR were observed in *Grey Mullet (Liza ramada)* fed on diets that contained zinc in nano form (Shukry, et al.,0.^2021^)*.* Similarly, Zhou et al. (2009) found a noticed increase in BWG and FW in crucian carp fed on a diet containing SeNPs, which indicated that nano-Se had different metabolic pathways. This theory was explained by Foster and Sumar (1995), who reported that Se bioavailability depended not only on its absorption by the intestine but also on its conversion to a biologically active form. However, nano-phosphorus could have a special metabolism pathway and deposition mechanism in Nile tilapia according to our results.

According to Deng and Chen (2003), nano-Se supplemented diet showed a significant increase in Nile tilapia (*Oreochromis niloticus*) growth rate. Ashouri et al. (2015) observed that nano-Se supplementation at different concentrations in common carp (Cyprinus carpio) diet turned into a noticed increase in growth performance parameters (FW and BWG). T. Muralisankar et al. (2014) noticed increased growth performance, activities, and survival rate in water prawn fish fed on a ZnNp diet. Khan et al. (2020) pointed out that feeding Nile tilapia on a nanoparticle-based nano-nutrient diet showed a higher growth rate. Furthermore, our results were compatible with these of Faiz et al. (2015), who assured that ZnO-NPs in juvenile grass carp (*C. idella*) fed promoted their growth performance. According to Alishahi et al. (2011), a significant increase in BWG and SGR was revealed in fish fed on ZnO-NPs diets compared to that fed on traditional ZnO diets at the same concentration. In rohu, *Labeo rohita* fingerlings showed a significant increase in growth rate higher than the control group when fed up with 20 mg/kg ZnO-NPs according to Mondal et al. (2020).

The nutritional state of fish regulates GHR and IGF-1, which together are considered good indicators for growth due to their effect on growth hormone (Amin et al. 2019; Pérez-Sánchez & Le Bail 1999). Our results pointed out that nano-phosphorus supplementation in the N-D group significantly upregulated GHR and IGF expression compared to the D-group (*p* < 0.05); meanwhile, their expression was non-significantly changed in the 1/2 N-D group (*p* ˃ 0.05). Similarly, Mahboub et al. (2020) noticed a significant upregulation in GH and IGF-1A hormones in African catfish (*Clarias gariepinus*) in groups fed on ZnO-NPs compared with others. Interestingly, Tawfik et al. (2017) found that nano ZnO increased the growth hormone in the serum of tilapia fish more than the conventional one (Zn oxide). Hoang Nghia Son and Le Thanh Long (2019) documented that the addition of nano mineral had an impressive effect on the expression of GHR and IGF in pigs. Furthermore, IGF-1 mRNA expression was enhanced in *goldfish (Carassius auratus)* fed on nanoparticles of iron (Akbary and Jahanbakhshi 2019). Moreover, Abd El-Kader et al. (2021) reported that European sea bass fed on a nano-selenium diet showed significant upregulation of GH and IGF-1 gene expression. In another trial on finishing pigs fed on a diet supplemented with chromium nanocomposite (CrNano), a clear increase in serum insulin-like growth factor-I was noticed, which coincided with a decrease in serum insulin and cortisol levels (Wang et al. 2007).

These results may be due to using of mineral with its nano form and this may change their characters. Additionally, the use of small nanoparticles induces better intestinal absorption and catalytic activities, which in turn improves their bioavailability (Awad et al. 2019). Also, Siklar et al. (2003) suggested that somatic growth enhances the DNA and RNA synthesis and promotes the synthesis of growth hormone protein. Supplementation of minerals in nano form has the ability to increase the surface of minerals for absorption and utilization (Vijayakumar and Balakrishnan 2014). Furthermore, nano form of minerals is often ranged between 1 and 100 nm, which improves the chance of absorption and makes it available through different body tissues and fluids; in addition, it induces their biological and physiological ability (Abd El-Kader et al (2021). This explanation is confirmed by Chen et al. (2006) who explained in detail many different ways by which nanoparticles work to increase the surface area and improve their interaction with biological support, including the increase of residency period in the gut and elimination of the intestinal clearance mechanisms.

Serum biochemical indices are a real mirror that shows the physiological processes and responses operate efficiently (Ismail et al. 2021; Hassaan et al. 2019). Monitoring of liver functions is one of the most effective ways to determine the impact of a certain substance on body healthiness (Mahboub & Shaheen 2021). Both SGOT and SGPT are major enzymes that give a clear picture on the condition of the liver, so their increases in serum or plasma are good proof that indicates a dysfunction or a defect in the liver (Ismail et al. 2022; Coz-Rakovac et al. 2005). In our study, SGPT liver enzyme showed an insignificant difference between groups, but SGOT enzyme showed a significant decrease in the N-D group with nano-phosphorus. This result is much close to a study reported by Deilamy et al. (2021) in Asian Sea bass juveniles fed on a diet supplemented by selenium and magnesium nanoparticles. That also was close enough to Ibrahim et al. (2021) who noticed that the lowest activities of SGOT were found in Nile tilapia fed on a diet containing selenium in nano form than other groups fed on the conventional one. In parallel to our findings, SGOT enzyme showed a significant decline in grass carp fed on a high-fat diet inlaid with nano-selenium compared with other groups (Liu et al., 2021). Moreover, Harsij et al. (2020) indicated that the lowest degree of SGOT was observed with increasing the dose of nano-selenium included in the rainbow trout diet. This could be attributed to the noticeable antioxidative effect, membrane stabilization, and cell damage prevention which led to better liver activity through adding of nanoparticles (Harsij et al. 2020). Similarly, Awad et al. (2019) suggested that the penetration ability of nanoparticles to liver cells enhances the production of cytokines that prevent liver injury. Meanwhile, others saw that liver enzymes (SGOT, SGPT) increased with the use of nanoparticles in rohu, *Labeo rohita* (Hamilton) fingerlings supplemented with Zn nanoparticle (Mondal et al. 2020). In contrast, there are researchers who found that there is no striking change in liver enzymes as a result of using nanoparticles (Ghazi, et al. 2021).

Our investigations revealed an increase of serum albumin in fish of the N-D group more than in other groups, which is compatible with Ghazi et al. (2021) on using selenium and zinc oxide nanoparticles. Additionally, using of nano-zinc oxide in Nile tilapia showed the same results (Awad et al. 2019). Also, there are much other papers that assured these results, such as Ashouria et al. (2015) in common carp, Abdel-Tawwab et al. (2007) in African catfish, and Dawood and Zommara (2020) in Nile tilapia. Swain et al. (2019) attributed such high levels of albumin and globulin to the effect of nanoparticles on the immunity. This may be due to diminished tissue damage and antioxidant capacity modulation of fish (Dawood et al. 2020; Kumar et al. 2018). It may also come back to the increased protein synthesis by the liver (Awad et al. 2019).

Several manuscripts have agreed that no noticeable change has been found in other biochemical analyses like creatinine or cholesterol and triglycerides by using nanoparticles, as reported by Hamed et al. (2021) in Asian sea bass fed on magnesium and selenium nanoparticles. In addition, no changes were found in creatinine level through using of nano zinc oxide in the diet of Nile tilapia (Awad et al. 2019). These results could be due to test duration, the size of the nanoparticle, the dose, and the type of nanoparticles (Hamed et al. 2021).

Our study revealed that the highest body composition of minerals such as Fe, Zn, P, and Ca was found in the N-D group compared with other groups. Regarding the amount of crude protein and fat content, they also were higher in the N-D group than in other groups; meanwhile, ash or moisture showed no significant differences between groups. Interestingly, carbohydrate showed its lowest value in the N-D group. Similar results were reported by Abdel-Hammed et al. (2019) in Nile tilapia fingerlings fed on nano iron and nano zinc diet. Prawns (*Macrobrachium rosenbergii*) post-larvae stage showed the best absorption of such minerals (Cu, Zn, K, Ca, Na, and Mg) when grew up on a diet supplemented with Fe2O3 nanoparticles (Srinivasan et al. 2016). Similarly, Bunglavan et al. (2014) assured the effect of nanoparticles on improving the bioavailability of the minerals. The study of Ahmed et al. (2021) confirmed that nanoparticle supplementation improved mineral absorption, crude protein, and fat content in *Cirrhinus mrigala* fingerlings fed on nano-selenium particles. Nile tilapia body composition showed a higher percentage of protein and Fe, Zn, and Cu when fed on nanoparticle-based nutrients (Khan et al. 2020). This may be due to either the special metabolism pathway by nanoparticles (Onuegbu et al. 2018; Zhou et al. 2009) or intestinal mineral antagonism reduction (Gopi et al. 2017).

The changes observed in lipid metabolism may be due to the change in mRNA levels of the genes related to it (Zhao et al. 2019). FAS is considered as a playmaker enzyme in the pathway called de novo lipogenesis (DNL) (Raza et al. 2018). *LPL* is one of the most important genes that are participated in the catabolism process of lipid (Tan et al. 2019) as it is responsible for the hydrolysis of lipoprotein triglycerides (Wang et al. 2008; Yan et al. 2015). In our study, genes related to fat metabolism such as FAS and LPL were significantly increased by nano-phosphorus partials supplementation at both concentrations, in opposite to the control group with traditional phosphorus. In accordance with our work, Liu et al. (2021) reported that dietary nano-Se addition to the diet of grass carp resulted in mRNA expressions elevation of LPL and regulation of the genes expression that related to the lipid metabolism. Moreover, this increase in *FAS* expression could be related to lipid metabolism improvement (Peng et al. 2021). Further investigation needs to be done in this regard.

There was a significant effect on the water phosphorus ratio due to the administration of different sources of phosphorus. Also, the largest percentage of phosphorous content was in fish of the N-D group, followed by the 1/2 N-D group; meanwhile, the percentage of water pollution by phosphorus was lower compared to the D-group. That may be due to better intestinal absorption of nanoparticles source so less excretion resulted, or nanoparticles minerals could inhibit mineral antagonism in the intestine, leading to excretion reduction and environment protection (Awad et al. 2019; Gopi et al (2017).

It could be concluded that using nano-phosphorus particles improved the growth rate and enhanced immunity response of tilapia fish, lowering the cost and protecting the environment.

## Data Availability

The data that support the findings of this study are available from the corresponding author upon request.
